# The Level of Knowledge about Toxoplasmosis among University Students in Rabat in Morocco

**DOI:** 10.1155/2021/5553977

**Published:** 2021-07-26

**Authors:** Sanaa Ait Hamou, Brahim Lamhamdi, Ichrak Hayah, Imane Belbacha, Abderrahim Sadak, Majda Laboudi

**Affiliations:** ^1^Laboratory of Ecology and Environment, Faculty of Science Ben M'Sik, Casablanca, Morocco; ^2^Faculty of Science Rabat, University Mohamed V, Rabat, Morocco; ^3^National Institute of Hygiene, Rabat, Morocco

## Abstract

The aim of this descriptive cross-sectional study is to evaluate the knowledge of toxoplasmosis among medical, biology, and veterinary students in Rabat in Morocco. The data was collected by using a questionnaire which includes demographic characteristics, epidemiology, diagnosis, and clinical issues related to knowledge of toxoplasmosis. During analysis, the study groups were divided based upon their specialty of students who were medical, biology, and veterinary students. Out of 230 students, 55.2% were female and 44.8% were male. The average age of the study population is 21.7 ± 02 years. Less than half (42.6%) have heard of the disease; most of them have heard from faculty during studies in classrooms with 75.8%, and 3.2% were from the internet. Only 36.5% knew the correct causative agent of toxoplasmosis, and 32.1% were aware of the definitive host. The current study documented that there are gaps in the knowledge of the students regarding toxoplasmosis. Therefore, the present study puts the basis for future studies highlighting the importance of educating students to improve knowledge and attitudes towards toxoplasmosis.

## 1. Introduction

Toxoplasmosis is a neglected zoonotic parasitic disease caused by an intracellular protozoan parasite: *Toxoplasma gondii* [1], an ubiquitous protozoan parasite which is ranked fourth among the 24 most harmful food-borne pathogens by the Food and Agriculture Organization (FAO) and the World Health Organization [2, 3]. Toxoplasmosis can infect all warm-blooded animals and humans [4]; it is mainly acquired by the oral ingestion of tissue cysts from undercooked meat containing bradyzoites or by ingestion of oocysts from food products (vegetables, fruit) or water contaminated by cats' feces containing sporozoites [5, 6].

Toxoplasmosis has a cosmopolitan distribution; approximately 30% of the world's human population has chronic toxoplasmosis [7]. However, seroprevalence varies from one country to another (6%-80%) and even within countries depending on many factors such as the differences in environment, eating habits, lifestyle, and hygiene of the populations and host susceptibility [8].

In spite of being one of the most common and widespread parasitic infections, toxoplasmosis is usually an asymptomatic and benign condition in immune-competent subjects [6]; it is still severe in immune-compromised patients and in cases of congenital infection [9]. This latter results from transplacental transfer of parasites from the mother to the fetus when there is a primary infection during pregnancy. The probability of transmission of the parasite to the fetus and the severity of the fetal attack evolve inversely during pregnancy [10]. Depending on the trimester of pregnancy, congenital infection can lead to abortion, hydrocephalus, retinochoroiditis, intracranial calcifications, or a psychomotor and neurological disability of the unborn child if the infection was in the first trimester [5, 11]. Furthermore, most infections acquired after birth cause mild symptoms such as ocular toxoplasmosis in immune-competent persons, or they remain asymptomatic [8]. On the other hand, in immune-compromised patients such as AIDS patients, a severe clinical manifestation due to reactivation of latent infections may occur and cause toxoplasmic encephalitis or death [5, 8].

Increasing the awareness of preventive measures for toxoplasmosis is considered the main approach in the prevention of toxoplasmosis because a vaccine for human toxoplasmosis is still under research and development [12]. Therefore, it is fundamental to educate students as future healthcare workers on various aspects of toxoplasmosis to resolve any doubts and to promote active awareness among the general population.

Previous studies have investigated the knowledge and perception of toxoplasmosis among university students in many countries worldwide. A study carried out in Iran revealed that only 15.7% of students are aware of toxoplasmosis [13]. In Iran, Nematollahi et al. reported that knowledge about the disease among students of a university is low [14]. A study performed in Malaysia on the knowledge of toxoplasmosis among female students reported a lack of knowledge of this disease in terms of primary prevention [15]. Another study conducted in Gulf countries such as Saudi Arabia revealed that a substantial proportion of the female student population did not have sufficient knowledge about the prevention of toxoplasmosis [16]. Moreover, in 2015, a study done on university students in Jordan reported the urgent need for awareness of toxoplasmosis and preventive education for women of reproductive age owing to insufficient knowledge of toxoplasmosis [17].

In Morocco, previous studies have reported a prevalence of toxoplasmosis among pregnant women and immunocompromised individuals [18–20]. These studies have documented that the prevalence is approximately 50% in pregnant women and that the main risk factors for toxoplasmosis among the target patient groups were insufficient knowledge of the disease and direct contact with soil [21].

According to the abovementioned prevalence rates in Morocco, more than half of women are not immunized and need sensitization and need to be sensitized to it and provided information regarding it to avoid congenital toxoplasmosis during pregnancy.

However, few studies have evaluated the knowledge of toxoplasmosis among health professionals and pregnant women [22, 23], and there are no studies on the knowledge of toxoplasmosis among university students. To our knowledge, our study is the first in the country to evaluate the knowledge about toxoplasmosis among university students of medicine, veterinary medicine, and biology in the Rabat prefecture in Morocco.

## 2. Subjects and Methods

This cross-sectional study was conducted in Rabat, north of Morocco, between 15 March and 15 June 2017; the target population consisted of medical, biology, and veterinary students selected in the Rabat area from the Faculty of Medicine and Pharmacy (FMP), Faculty of Sciences (FS), and Institute of Agronomic Veterinary Hassan II (IAV).

Regarding the impossibility of identifying all the student targets, the recruitment of our study population was based on a simple random sampling at the level of each faculty. In addition, recruitment of university students from all years of study was conducted through collaboration with the deanship of students' affairs at each faculty.

We estimated the sample size using the formula recommended by the World Health Organization [24]. The following criteria were established: the adequate knowledge rate at 50%, the confidence level at 95%, and the margin of error at 5%. The total number involved in this study was 230 according to the following criteria: students who accepted to participate in this study and completed the questionnaire.

Data were collected using a structured self-administered questionnaire designed to evaluate the knowledge of students on toxoplasmosis. The questionnaire was pilot tested in 2017 in 30 students to assess its clarity and validity, and subsequently, adjustments were made where necessary. The content validity was assessed by a public health expert and a professor of parasitology, and subsequently, the necessary adjustments were made. Students who participated in the pilot study were excluded from the final analysis.

The questionnaire consisted of two main parts; demographic information includes age, sex, educational level, and having heard about toxoplasmosis disease. And the second section comprised the epidemiology, diagnosis aspects, and clinical manifestations of toxoplasmosis knowledge. Data were collected from medical students attending the 1st to 5th years, biology students attending the1st to 3rd years, veterinary students attending the 1st to 5th years, master students, and PhD students.

During the statistical analysis, the study groups were divided based upon their specialty of students who were medical, biologist, and veterinary students.

All the data from the questionnaires were put together in Epi Info software 2012 version (3.5.4). Descriptive statistics using frequencies and percentages were used to identify participants' knowledge about toxoplasmosis. A chi-squared (*χ*^2^) test was performed to examine the association among the categorical variables including the relationships between the different characteristics of participants with some variables included in the questionnaire. The results will be considered statistically significant when *p* < 0.05.

All participants were briefed about the study objectives, and written informed consent was obtained. Confidentiality and privacy were assured and maintained throughout the study.

## 3. Results

### 3.1. Sociodemographic Characteristics of the University Students

A total of 230 students from three government schools in Rabat participated in the study; the average age is 21.7 ± 2 years, of whom 44.8% (103/230) are males and 55.2% (127/230) were females. Most of the students (58.3%) were in the age group between 21 and 23 years.

Regarding the distribution of the study population, 31.3% were medical students from FMP, 50% were biology students from FS, and 18.7% were veterinary students from IAV (Table 1).

Among the two hundred thirteen respondents interviewed during the study, 42.6% of the students were aware (having heard or read) about toxoplasmosis while 57.4% were unaware of this protozoan parasite (Table 1), of which 47.2% were female and 36.9% were male. However, this difference is not statistically significant. Most of the students who have heard of the disease belong to the age group of 21-23 years (49.7%), while students aged over 24 years and under 20 years have only 42.4% and 28.6%, respectively; this result is statistically significant (Table 1).

75.8% reported that they had information during their faculty studies, while 24.3% had heard of toxoplasmosis via internet, media, and reading. Their proportions are, respectively, 17.9%, 3.2%, and 3.2%. The least important source was from reading (3.2%). Further, our results revealed that there was a statistically significant positive correlation between the level of awareness about toxoplasmosis and the level of education of participants' answers about having heard of the disease. We found that the undergraduate biology and veterinary students and PhD level knew less about the disease than the master degree and undergraduate medical students' level (*p* < 0.05) (Table 1).

### 3.2. Knowledge of Agent Infection and Epidemiology of Toxoplasmosis

Of the total population of students, nearly 36.5% were aware that toxoplasmosis is caused by the parasite *T. gondii* and 32.1% were aware that the definitive host is a cat. Most of the students who have responded correctly were medical and biology students belonging to the FMP and FS, respectively, while less than 25% of the veterinary students are part of the IAV (Table 2).

Concerning the knowledge of risk factors for toxoplasmosis, thirty per cent of students answered correctly that the main risk factor for transmission of the parasite is oral food by ingestion of undercooked meat or food product contaminated with cat feces or direct contact with a cat. On the other hand, only 13.9% answered correctly that untreated water can transmit the parasite of the disease (Table 2).

### 3.3. Knowledge of Diagnosis and Clinical Manifestation of Toxoplasmosis

Among the university students interviewed about the diagnosis of toxoplasmosis, 29.5% knew that the diagnosis of toxoplasmosis is mainly based on research of IgG and IgM *T. gondii* antibodies and only 23.9% knew that the research of IgM T. gondii antibody could be used to estimate the toxoplasmosis infection during pregnancy. Besides, 84% of students did not know the avidity test. On the other hand, only 14.7% correctly answered that there is no vaccine for humans available to the public that will protect a person from getting toxoplasmosis (Table 2).

Regarding the clinical manifestation knowledge, 18.7% of students knew seroconversion while 32.6% respond correctly about the definition of congenital toxoplasmosis. Besides, 16.9% reported the correct answer that toxoplasmosis in most cases is an asymptomatic disease. However, incorrect answers about the knowledge of the highest gravity time are more than 15%. Further, less than half (26.6%) of participants knew that toxoplasmosis is an opportunistic disease in immunocompromised persons (Table 2).

## 4. Discussion

Improving knowledge and awareness of toxoplasmosis is considered to be the most important factor in preventing the risk of congenital toxoplasmosis. Our study was undertaken to evaluate the level of knowledge of toxoplasmosis among students of medicine, biology, and veterinary medicine. Hence, the percentage of university students who were aware of toxoplasmosis was 42.6%, which was higher than that observed in Iran (15.7%) [13], in Saudi Arabia (28%) [16] and (20.9%) [25], and recently in Egypt (3.2%) [26]. However, our finding is lesser than that reported in Yemen (50%) [15] and in Jordan (51.1%) [17]. The different target populations in the studies and the different cultural and economic contexts of each country may explain this variation in the knowledge of the disease.

Most of the students (75.8%) who are previously aware about toxoplasmosis reported that they had acquired this knowledge in the classroom during their study. Similar previous studies have reported differences in the source of information. In Yemen, Al-naggar et al. (2010) reported that half of the students in the sample knew about toxoplasmosis through the internet [15]. Similarly, a study in Saudi Arabia reported that more than half of the respondents obtained their information from the internet (55.5%) [27]. In contrast, a study done in Iran in 2013 reported that the most important source of information for the participants was books (55.8%) [13].

We also investigated the relationship between gender and the knowledge of the students about the disease. More female students (47.2%) than male students (36.9%) are aware about the disease. In contrast, previous studies have reported an opposite trend. Nematollahi et al. in 2011 noted that the knowledge rate of male students was higher than that of female students (41% and 13.9%), respectively [14]. Similarly, Ebrahimi et al. (2015) reported that more male students were aware of the disease (17.3%) than female students (13.8%) [13]. These results may be explained by the cultural context of each country, which is different from that of Morocco.

Regarding the relationship between the level of study and the knowledge of students about the disease, we found that medical students at the master's and undergraduate levels were more aware of toxoplasmosis than those at other educational levels. These results are not in agreement with the findings reported by Ebrahimi et al. which suggest that only around 10% of the participants reported that PhD students knew more about the disease than the students in their first year of study in the faculty [13]. This discrepancy may be due to the course content at the master's level, which is rich in parasitology-related information.

In the present study, 36.5% of students stated that toxoplasmosis is caused by the parasite *T. gondii*, and 32.1% of the students were aware of the definitive host (cat). These percentages are lower than those reported in the studies carried out in Yemen and Iran, in which 50% of the students knew that toxoplasmosis is a parasitic disease transmitted by *T. gondii* and 64% were aware that the definitive host is cat [13, 15]. In contrast, in Saudi Arabia, only 25.1% and 50.25% of the students, respectively, knew the causative agent and the role of cats in the transmission of toxoplasmosis [27].

We also compared the knowledge and perception of students on the different routes of transmission of toxoplasmosis and observed a variation in the knowledge level among various university students. Approximately 30% of students correctly indicated that transmission of the parasite mainly occurs through the oral route, and the main risk factors include ingestion of undercooked meat or food products contaminated with cat feces and direct contact with cats. This finding is lower compared to that reported in many studies conducted in other countries. In Iran, approximately 54% of university students correctly answered that the oral route is the most important for the transmission of toxoplasmosis [13, 15]. Another study undertaken in Iran among female students showed that 72.5% correctly believed that the consumption of undercooked meat is an important route of transmission [28]. In 2000, Cook et al. reported that the dietary route is the most important for the transmission of toxoplasmosis [29].

Furthermore, few university students (33%) mentioned that cats are the main source of environmental contamination. Another study conducted in Iran reported that only 19.4% of respondents correctly identified contact with cat feces as the main mode of transmission of toxoplasmosis [30]. Indeed, climatic factors play a major role in the survival of oocysts in the environment in addition to their role in the infection rates in meat-producing animals. Tropical countries with a humid and warm climate typically show a higher prevalence of toxoplasmosis than countries with an arid or cold climate. [31].

In the present study, few students (13.9%) believed that toxoplasmosis may be transmitted through untreated water. A similar belief has also been reported in other parts of the world. Alrashada et al. reported that 51.3% of female students knew that drinking tank water is a risk factor for toxoplasmosis transmission [16]. A study performed in France in 2004 reported a positive correlation between the consumption of unboiled water and the presence of anti-*T. gondii* antibodies, particularly at farms with poor hygienic conditions and shallow wells [32]. Until recently, the presence of *T. gondii* in public drinking water was reported in Mexico. Toxoplasmosis may be acquired through the ingestion of drinking water contaminated with oocysts of *T. gondii*, which are highly resistant to the standard disinfection processes, such as chlorination, commonly used in the water supply industry [33].

Approximately 24% of students interviewed in the present study mentioned that the diagnosis of toxoplasmosis is mainly based on the detection of anti-*T. gondii* IgG and IgM antibodies, and only 29.5% of students knew that the avidity test is a test for dating the infection during the first months of pregnancy. Indeed, detection of IgM is not always considered sufficient evidence of recent infection [34]. Nevertheless, the avidity test appears to be effective and reliable, allowing for the exclusion of active toxoplasmosis [35]. Laboudi and Sadak who conducted a study in the Rabat region of Morocco showed that investigation of IgM-positive serum samples with the avidity test is helpful in eliminating recently acquired toxoplasmosis infection in pregnant women during their first trimester of pregnancy [36].

In response to the questions about the existence of a vaccine for human toxoplasmosis, only 14.7% of university students answered correctly that a vaccine for humans, which will protect an individual from getting toxoplasmosis, is not available to the public. This percentage is lower than that reported by Ansari-lari et al.; in their study, 46% of female university students believed that the disease is preventable by vaccination [28]. This prevention method is theoretically conceivable because of the strong immune response observed during natural infection. Vaccines for cats or other animals are a noteworthy option for reducing the parasite load in the environment. In sheep, a live attenuated vaccine for toxoplasmosis is marketed [37] mainly for the prevention of toxoplasmic abortions. In the absence of an effective vaccine in humans, following the best preventive practices may be the optimal approach to managing toxoplasmosis and must be done by limiting exposure to oocysts or tissue cysts.

The present study indicated that 18.7% of students knew what seroconversion is, whereas 32.6% were aware of the definition of congenital toxoplasmosis. These findings suggest that most students are confused between the concepts of seroconversion and congenital toxoplasmosis. Seroconversion occurs when pregnant women develop IgG antibodies to *T. gondii*; it is characterized by a significant increase in IgG antibody titers together with the presence of IgM antibodies. However, congenital toxoplasmosis is defined as the transmission of the parasite from mother to child during pregnancy [37].

Unfortunately, most participants in the present study seemed to misunderstand the period of highest transmission and highest severity during pregnancy that affects the fetus. Approximately 14% of the respondents answered correctly about the period of highest transmission and severity during the different trimesters of pregnancy. Indeed, the highest rate of transmission is observed during the third trimester, and the highest severity, if the parasite is transmitted to the fetus, is observed in the first trimester because the placental barrier is thicker during the first trimester and becomes thinner towards the end of the pregnancy [37, 38].

Less than half of the respondents knew that toxoplasmosis is an opportunistic disease in immunocompromised individuals. Although toxoplasmosis infection is asymptomatic in immunocompetent individuals, it leads to serious pathological effects in immunodeficient patients [39]. It has emerged as a major opportunistic disease in patients with acquired immunodeficiency syndrome. It can manifest as potentially fatal encephalitis owing to the reactivation of latent infections in HIV-associated immune suppression [40].

## 5. Limitations of the Study

Although the results do not represent all students in Rabat, it gives an insight into one group of undergraduates in Morocco regarding their knowledge towards toxoplasmosis. However, there was one limitation in the current study; the sample size was not as high as expected. The low sample size could be explained by the fact that this study was done and data was collected during the time of exams for university students.

## 6. Conclusion

The current study proved the low level of knowledge about toxoplasmosis among university students in Rabat. Therefore, this study reflects the necessity to educate students about the knowledge and hygienic measures which are essential to avoid infection. This is better done through adopting health education programs to educate people in groups, such as in universities, hospitals, and other work areas. Likewise, it is recommended that other studies should be repeated with a larger sample size to include other faculties.

## Figures and Tables

**Table 1:** Relationship between explanatory variables as demographic data and having heard about toxoplasmosis among university students in Rabat prefecture in Morocco.

Variables	Students aware about toxoplasmosis	Students unaware about toxoplasmosis	*χ* ^2^	*p*
Sex				
Female	60 (47.2%)	67 (52.8%)	2.49	0.114
Male	38 (36.9%)	65 (63.1%)		
Age				
≤20 years	18 (28.6%)	45 (71.4%)	7.49	*p* < 0.05^∗^
21-23 years	66 (49.3%)	68 (50.7%)		
≥24 years	14 (42.4%)	19 (57.6%)		
Level of education				
Undergraduate medical students	42 (59.2%)	29 (40.8%)	21.54	*p* < 0.05^∗^
Undergraduate biology students	28 (30.1%)	65 (69.9%)		
Undergraduate veterinary students	14 (32.6%)	29 (67.4%)		
MSc	11 (73.3%)	4 (26.7%)		
PhD	3 (37.5%)	5 (62.5%)		
Population target/name of faculty				
Medical students/FMP	43 (59.7%)	29 (40.3%)	12.67	*p* < 0.05^∗^
Biology students/FS	41 (35.7%)	74 (64.3)		
Veterinary students/IAV	14 (32.6%)	29 (67.4%)		

^∗^Statistically significant.

**Table 2:** Answers to knowledge about the infectious agent and epidemiology, diagnosis, and clinical aspects of toxoplasmosis among university students in Rabat prefecture in Morocco.

Variables	Correct responses, *N* (%)			
	Medical students (*n* = 72)	Biology students (*n* = 115)	Veterinary student (*n* = 43)	Total (*N* = 230)
The knowledge of agent infection	38 (52.8%)	35 (30.4%)	11 (25.5%)	84 (36.5%)
The knowledge about host definitive	36 (50%)	29 (25.2%)	9 (20.9%)	74 (32.1%)
The main of toxoplasmosis transmission is oral food route	32 (44.4%)	29 (25.2%)	8 (18.6%)	69 (30%)
Undercooked meat	7 (9.8%)	33 (28.6%)	7 (16.2%)	74 (32.1%)
Untreated water	16 (22.2%)	12 (10.4%)	4 (9.3%)	32 (13.9%)
Food product with cat's feces	33 (45.9%)	32 (27.8%)	12 (27.9%)	77 (33.4%)
Contact direct with cat	35 (48.7%)	29 (25.2%)	12 (27.9%)	76 (33%)
Research of IgG and IgM *T. gondii* antibodies	30 (41.7%)	29 (25.2%)	9 (20.9%)	68 (29.5%)
Role of IgM *T. gondii* antibody determination of toxoplasmosis	25 (34.8%)	23 (20%)	7 (16.2%)	55 (23.9%)
Knowledge of avidity test	20 (27.8%)	13 (11.3%)	4 (9.3%)	37 (16%)
Existence of human vaccine toxoplasmosis	13 (18%)	17 (14.7%)	4 (9.3%)	34 (14.7%)
Definition of seroconversion	24 (33.3%)	14 (12.1%)	5 (11.6%)	43 (18.7%)
The knowledge of congenital toxoplasmosis definition	36 (50%)	28 (24.3%)	11 (25.5%)	75 (32.6%)
Most cases of toxoplasmosis are asymptomatic	21 (29.1)	13 (11.3%)	5 (11.6%)	39 (16.9%)
Opportunist disease in immunocompromised persons	31 (43.0%)	2 (1.7%)	8 (18.6%)	61 (26.6%)
The high risk period of transmission in the third trimester of pregnancy	22 (30.6%)	9 (7.8%)	2 (4.6%)	33 (14.3%)
The high gravity period of lesion during the first trimester of pregnancy	21 (29.1%)	7 (6.6%)	4 (9.3%)	32 (13.9%)

## Data Availability

The datasets used during the current study are available from the corresponding author on reasonable request.
